# Exercise-Induced Neuroprotection of Hippocampus in APP/PS1 Transgenic Mice via Upregulation of Mitochondrial 8-Oxoguanine DNA Glycosylase

**DOI:** 10.1155/2014/834502

**Published:** 2014-11-05

**Authors:** Hai Bo, Weimin Kang, Ning Jiang, Xun Wang, Yong Zhang, Li Li Ji

**Affiliations:** ^1^Tianjin Key Laboratory of Exercise Physiology and Sports Medicine, Department of Health & Exercise Science, Tianjin University of Sport, Tianjin 300381, China; ^2^Department of Military Training Medicines, Logistics University of Chinese People's Armed Police Force, Tianjin 300162, China; ^3^Department of Interventional Neuroradiology, Chinese People's Second Artillery General Hospital, Beijing 100080, China; ^4^Laboratory of Physiological Hygiene and Exercise Science, School of Kinesiology, University of Minnesota, Minneapolis, MN 55455, USA

## Abstract

Improving mitochondrial function has been proposed as a reasonable therapeutic strategy to reduce amyloid-*β* (A*β*) load and to modify the progression of Alzheimer's disease (AD). However, the relationship between mitochondrial adaptation and brain neuroprotection caused by physical exercise in AD is poorly understood. This study was undertaken to investigate the effects of long-term treadmill exercise on mitochondrial 8-oxoguanine DNA glycosylase-1 (OGG1) level, mtDNA oxidative damage, and mitochondrial function in the hippocampus of APP/PS1 transgenic mouse model of AD. In the present study, twenty weeks of treadmill training significantly improved the cognitive function and reduced the expression of A*β*-42 in APP/PS1 transgenic (Tg) mice. Training also ameliorated mitochondrial respiratory function by increasing the complexes I, and IV and ATP synthase activities, whereas it attenuated ROS generation and mtDNA oxidative damage in Tg mice. Furthermore, the impaired mitochondrial antioxidant enzymes and mitochondrial OGG1 activities seen in Tg mice were restored with training. Acetylation level of mitochondrial OGG1 and MnSOD was markedly suppressed in Tg mice after exercise training, in parallel with increased level of SIRT3. These findings suggest that exercise training could increase mtDNA repair capacity in the mouse hippocampus, which in turn would result in protection against AD-related mitochondrial dysfunction and phenotypic deterioration.

## 1. Introduction

Alzheimer's disease (AD) is a neurodegenerative disorder characterized by the progressive loss of memory and cognitive function. One of the pathological hallmarks of AD is abnormal accumulation of amyloid-*β* peptide (A*β*), derived from the amyloid precursor protein (APP) via proteolytic cleavages by *β*- and *γ*-secretases [1]. Mitochondrial dysfunction and oxidative stress have been observed in the postmortem brains of AD transgenic mice and in cell lines that express mutant APP [2, 3]. Multiple lines of evidence suggest that age-dependent production of A*β* triggers mitochondrial dysfunction through a number of potential pathways such as impaired oxidative phosphorylation, increased reactive oxygen species (ROS) production, altered mitochondrial dynamics, and their interactions with the mitochondrial components [4].

On the other hand, accumulating research data indicate that mitochondrial bioenergetic homeostasis and probably brain metabolism in general can affect A*β* deposition, synaptic degeneration, and neurofibrillary tangle formation in AD [5]. In particular, mitochondria-derived ROS are sufficient to trigger amyloidogenic APP-processing in vitro and in vivo [2]. Based on these findings, it was proposed that A*β* could cause mitochondrial malfunction and oxidative stress, which in a vicious cycle reinforces A*β* deposition. Since mitochondrial dysfunction is considered as a major functionally relevant pathogenic mechanism for AD, improving mitochondrial function has been proposed as a reasonable therapeutic strategy to reduce A*β* load and to modify the progression of the disease [6].

Mitochondrial DNA (mtDNA) has a closed circular structure and is located near the mitochondrial electron transport chain (ETC), but it lacks histones, which protect the DNA against oxidative modification by ROS. Under oxidative stress, the bases of mtDNA are susceptible to oxidation to 8-oxo-deoxyguanosine (8-oxodG). In frontal, parietal, and temporal lobes of AD brain, the level of 8-oxodG is significantly elevated, and the level of 8-oxodG in mtDNA is approximately 10-fold higher than that in nuclear DNA [7]. Although no clear causative mutations in the mtDNA have been linked to AD, it has been shown that oxidative damage in mtDNA could cause energy deficit, increased oxidative stress, and accumulation of A*β*, all potential contributors to the pathogenesis of AD [1].

Concomitant to ROS production, an inefficient mitochondrial base excision repair (BER) machinery has also been pointed to favoring the accumulation of oxidized bases in mtDNA during AD progression [8]. Although a number of subpathways exist, 8-oxoguanine DNA glycosylase-1 (OGG1), which is primarily responsible for removing 8-oxodG from DNA, is thought to play a central role in the BER system [9]. Shao et al. [10] demonstrated that mitochondrial OGG1 activity decreased in the frontal and temporal lobe of late-stage AD and in the temporal lobe of the mild cognitive impairment (MCI) patients, compromising the removal of oxidatively damaged bases from mtDNA. Thus, a downregulation of mitochondrial OGG1 may play an important role in the pathogenesis of AD.

Exercise training is considered to be one of the effective interventions for ameliorating the pathological phenotypes of AD. Neuronal mitochondrial resilience, respiratory properties, and antioxidant defense have been reported to increase after endurance exercise, resulting in increased resistance to various stresses, such as anoxia, ischemia-reperfusion, and neurotoxins [6]. However, to our knowledge, few studies have investigated the relationship between mitochondrial adaptation and brain neuroprotection caused by physical exercise in AD. Furthermore, it has been shown that exercise training can improve mitochondrial OGG1 level and activity in skeletal muscle and liver [11, 12]. Thus, in the present study we used an APP/PS1 transgenic mouse model of AD to test the hypothesis that exercise training could increase mtDNA repair capacity in the mouse hippocampus, which in turn would result in protection against AD-related mitochondrial dysfunction, A*β* deposition, and cognitive functional decline.

## 2. Methods

### 2.1. Animals and Treatment

Male double-transgenic mice (APP/PS1 mice) expressing a chimeric mouse/human APP (Mo/HuAPP695swe) and a mutant human presenilin 1 (PSl-ΔE9) and their wile-type littermates were obtained from the Institute of Laboratory Animal Science, Chinese Academy of Medical Science (Beijing, China). APP/PS1 Tg mice have accelerated the AD phenotype characterized by increased A*β* deposits and behavioral deficits as young as 13–16 weeks [13]. The animals were housed in double cages in a temperature-controlled room (21-22°C; 50–60% humidity) with a 12 h light/12 h dark cycle and were provided with free access to a chow food and tap water. The animal experimental protocols conformed to the Chinese Guidelines for the Care and Use of Laboratory Animals. At the age of 3 months, the mice were randomly divided into four groups: sedentary wild-type mice (SED-Wt, *n* = 32), exercise-trained wild-type mice (EXE-Wt,  *n* = 32), sedentary APP/PS1 transgenic mice (SED-Tg,  *n* = 32), and exercise-trained APP/PS1 transgenic mice (EXE-Tg,  *n* = 32).

The mice in the EXE-Wt and EXE-Tg group were allowed to adapt to treadmill running for 10 min each day on 3 consecutive days at 8 m/min, 0% grade. After acclimatization, all of the exercise animals were subjected to running for 30 min a day at 11 m/min and 0% grade for 5 days/week for 20 weeks. Only gentle tail touching was used to prompt the mice to run to minimize the stress associated with treadmill exercise. This protocol was shown to improve cognitive function and alter neurotrophic factor levels in the APP/PS1 mice [14]. The SED-Wt and SED-Tg groups of mice were placed on the treadmill without running for the same period as the EXE mice, such that they were exposed to potential handing and environment stresses without producing a training effect. Behavioral studies and biochemical analyses were carried out in a double-blind manner such that the operator was not aware of the label of the treatment groups.

### 2.2. Passive Avoidance Test

Learning ability and memory were evaluated using a passive avoidance test apparatus 24 h after the last exercise session. The apparatus consisted of a light compartment connected by a guillotine door to a dark compartment with a stainless steel electric shocking grid floor. On the first day, the mouse was placed into the light compartment facing the wall and received a mild footshock (0.5 mA, 1 s) immediately as it stepped into the dark compartment. The time that a mouse stayed in the light compartment was recorded as the preshock retention latency. On the next day, the animal was again placed into the light compartment, and the time that the animal stayed in the light compartment before stepping into the dark compartment was recorded as the escape latency. The ceiling score was chosen to be 300 s.

### 2.3. Y-Maze Test

We examined continuous spontaneous alternation behavior using the Y-maze apparatus. The Y-maze apparatus was made of black plastic with three arms (40 cm long, 3 cm wide, and 12 cm high) extending from a central platform at 120°. Mice began a single trial at the end of one arm and were allowed to freely explore the Y-maze for 8 min. Arm entry was defined as the entry of 4 paws into one arm. The number and sequence of arm entries were recorded manually by an observer. Alternation was defined as a consecutive entry in three different arms. The percentage of spontaneous alternation was calculated with the following formula: [(number of alternations)/(total arm entries − 2)] × 100.

### 2.4. Isolation of Mitochondria

Hippocampus mitochondria were isolated as described previously [15]. Following the passive avoidance test, mice were sacrificed by decapitation, the brains were removed, and the hippocampus was dissected on ice. Hippocampus from four mice in one group was pooled to obtain sufficient amount of protein for each group for biochemical analysis. Tissues were homogenized in an ice-cold isolation buffer containing 230 mM mannitol, 70 mM sucrose, 1 mM EDTA, and 10 mM Tris-HCl (pH 7.4) with a weight/volume ratio of 1 : 10. The homogenate was centrifuged at 700 g for 10 min, followed by centrifugation of the supernatant at 8,000 g for 10 min to obtain a mitochondrial pellet. The pellet was washed twice and resuspended in isolation medium containing 15% Percoll and layered on preformed Percoll gradients (40 and 23%). The gradient was spun at 30,700 g for 10 min, and the 23/40 interphase layer, containing the mitochondria, was collected. The mitochondria fraction was diluted with isolation buffer and centrifuged at 16, 700 g for 12 min. The pellet was then washed with isolation buffer containing bovine serum albumin (BSA) and centrifuged at 9,800 g for 10 min. The pellet containing purified mitochondria was resuspended in 100 *μ*L of isolation buffer without EDTA. Mitochondrial protein content was assayed using a BCA protein assay reagent kit (Thermo Scientific, USA).

### 2.5. Mitochondrial Respiration

Mitochondrial respiratory function was measured polarographically at 25°C using an Oxygraph system (Hansatech Instruments) [16]. After electrode calibration, isolated mitochondria (300 *μ*g) were added to 0.5 mL of respiration buffer (130 mM KCL, 3.0 mM Hepes, 0.5 mM EDTA, 2.0 mM KH_2_PO_4_, and 1 mg/mL BSA, pH 7.4). State 2 respiration was started with the addition of 1 mM glutamate and 0.1 mM malate. After stable state 2 respiration was established, state 3 respiration was initiated by the addition of 100 nM ADP. When all of the ADP added had been phosphorylated to ATP, the respiratory rate returned to state 4. The respiratory control ratio (RCR) was calculated as the ratio of the respiratory rate in state 3 to that in state 4.

### 2.6. Mitochondrial ROS Production

Mitochondrial ROS generation was monitored in fresh mitochondrial suspensions using the dichlorofluorescin diacetate (H_2_-DCFDA) probe [16]. Briefly, the H_2_-DCFDA stock solution was dissolved in 1.25 mM methanol and kept in the dark at 0°C. To initiate the experiment, 250 *μ*g hippocampal mitochondria were added to a quartz cuvette containing 2 mL of 0.1 M phosphate buffer (pH 7.4) and 2 *μ*L of 2.5 mM H_2_-DCFDA (total volume 0.3 mL). The assay mixture was incubated at 37°C for 15 min to allow the H_2_-DCFDA probe to enter the mitochondria. DCF formation was determined fluorometrically at the excitation wavelength of 499 nm and emission wavelength of 521 nm at 37°C for 2 min, using a Cary Eclipse Fluorescence spectrophotometer (Varian, Santa Clara, CA, USA). A blank consisting of the appropriate buffer and 5.0 *μ*M H_2_-DCFDA without mitochondria was used to correct for the autoxidation rate of H_2_-DCFDA. The units are expressed as pmol DCF formed per minute per milligram of protein.

### 2.7. Mitochondrial Respiratory Complex Activity

Mitochondria were treated with 1 mg cholate/mg mitochondrial protein and further ruptured by one cycle of freeze-thaw (−80°C/25°C). Mitochondrial respiratory complex activities were measured spectrophotometrically as specific donor-acceptor oxidoreductase activities according to Shi et al. [17]. The enzyme activity was expressed as nanomoles per min per milligram of protein.

Complex I (NADH-ubiquinone oxidoreductase) activity was measured by following the decrease in absorbance due to the oxidation of NADH at 600 nm. NADH (0.1 mM), ubiquinone (0.1 mM), antimycin A (3 *μ*M), and mitochondria (30 *μ*g) were added to the assay medium (25 mM potassium phosphate, 5 mM MgCl_2_, 2 mM KCN, pH 7.2) and the absorbance change was recorded for 5 min.

Complex II (succinate-ubiquinone oxidoreductase) activity was performed at 600 nm using dichlorophenolindophenol (DCPIP) as acceptor and succinate as donor. The reaction mixture containing 30 *μ*g mitochondria, 25 mM potassium phosphate, 5 mM MgCl_2_, 2 mM KCN, 20 mM succinate, 3 *μ*M antimycin A, 2 mM KCN, 1 *μ*M rotenone, and 0.1 mM DCPIP was preincubated for 10 minutes at 37°C to minimize the succinate dependent nonlinear rate. The reaction was started with ubiquinone (0.1 mM) and the enzyme-catalyzed reduction of DCPIP was recorded for 5 min.

Complex III (ubiquinone-ferricytochrome-c oxidoreductase) activity was measured by monitoring the reduction of cytochrome c at 550 nm in the assay medium (25 mM potassium phosphate, 5 mM MgCl_2_, 2 mM KCN, 2 *μ*g/mL rotenone, pH 7.2). Cytochrome c (15 *μ*M) and ubiquinol (0.1 mM) were added to the assay medium, and the nonenzymatic rate was recorded for 1 min. Then, mitochondria were added, and the increase in absorbance was recorded for 3 min.

Complex IV (cytochrome c oxidase, COX) activity was measured as the oxidation of reduced cytochrome c. The assay mixture contained 40 *μ*M reduced cytochrome c in the assay medium containing 20 mM potassium phosphate, pH 7.0. The reaction was started by the addition of mitochondria and absorbance change was recorded at 550 nm for 2 min.

### 2.8. Mitochondrial ATP Synthase Activity

Mitochondrial ATP synthase activity was determined using a bioluminescence technique [16]. Mitochondrial suspensions were added to a cuvette containing 0.1 M luciferase, 0.25 M sucrose, 3.0 mM Hepes, 0.5 mM EDTA, and 0.1 mM pyruvate +1 mM malate as substrate. After background bioluminescence was determined for correction, 2 *μ*M of ADP was added to initiate the reaction. ATP production was monitored at 25°C with a BioOrbit 20/20^*n*^ luminometer (Turku, Finland) and expressed as nmol/sec per mg protein.

### 2.9. Mitochondrial Antioxidant Enzymes Activity

Mitochondrial Mn-superoxide dismutase (MnSOD) activity was measured using the method of McCord and Fridovich [18]. MnSOD activity in solubilized mitochondrial extracts was measured by the cytochrome c reduction method in the presence of 1 mM KCN to inhibit both Cu-Zn SOD and extracellular SOD. The amount of enzyme required to produce 50% inhibition was taken as one unit and results were expressed as U/mg protein.

Mitochondrial glutathione peroxidase (GPx) activity was measured according to the method described by Wendel [19] using tert-butyl hydroperoxide as substrate. NADPH disappearance was monitored spectrophotometrically at 340 nm in the reaction mixture containing 20 *μ*g mitochondria, 2 mM glutathione, 0.15 U/mL glutathione reductase, 0.4 mM azide, 0.5 mM tert-butyl hydroperoxide, and 0.1 mM NADPH. One GPx unit is defined as 1 *μ*mol of NADPH consumed per minute and the specific activity is represented as units/mg protein.

### 2.10. Measurement of 8-oxodG in mtDNA

Mitochondrial DNA separation and 8-oxodG measurement were performed as described previously [11]. Briefly, mtDNA was purified by the mtDNA Extractor CT kit according to the recommended protocol (Wako Pure Chemical Co., Japan). The mtDNA extraction procedure was optimized to minimize artificial induction of 8-oxodG by using radical-free phenol, minimizing exposure to oxygen, and by the addition of 1 mM deferoxamine mesylate and 20 mM TEMPO (2,2,6,6-tetramethylpiperidine-N-oxyl), according to the European Standards Committee on Oxidative DNA Damage [20]. Purified mtDNA was digested to deoxynucleotides by incubation at 37°C with 5 U nuclease P1 (in 20 *μ*L of 20 mM sodium acetate, 10 mM ZnCl_2_, 15% glycerol, pH 4.8) for 30 min and with 1 U of alkaline phosphatase (in 20 *μ*L of 1 M Tris-HCl, pH 8.0) for 1 h. The 8-oxodG and dG concentrations were measured by HPLC with online electrochemical and ultraviolet detection, respectively. After filtering through a 0.22 *μ*m filter membrane (Millipore, Bedford, MA, USA), the reaction mixture was applied to a HPLC system with a symmetry C18 column (Waters Associates, Milford, MA, USA) that was attached to a Coulochem electrochemical detector (ESA, Bedford, MA, USA) to measure 8-oxodG. An HP 1100 series UV detector at 254 nm was used to measure dG. The levels of 8-oxodG are expressed as the molar ratio of 8-oxodG to 10^5^ dG.

### 2.11. Analysis of Mitochondrial OGG1 Activity

Hippocampus mitochondrial OGG1 activity was determined in fresh mitochondrial suspensions by incision assays according to Radak et al. [12]. Briefly, an 8-oxodG-containing single stranded 24-mer oligonucleotide was labeled at the 5′end with [*γ*-^32^P] ATP using T4 polynucleotide kinase and was then hybridized with the complementary oligonucleotide by incubation at 90°C for 10 min, followed by gradual cooling to room temperature. After permeabilization in the presence of 0.05% Triton X-100 and 0.3 M KCl, 20 *μ*g of mitochondria was incubated at 37°C for 1 h in reaction mixture containing 40 mM Hepes-KOH (pH 7.6), 5 mM EDTA, 2 mM DTT, 75 mM KCl, 10% glycerol, and 88.7 fmol ^32^P-labeled duplex oligonucleotide. 10 mM NaOH was added, and the mixture was further incubated for 15 min at 37°C and the reaction was then stopped by adding 4 *μ*L of formamide dye, followed by heating for 5 min at 95°C. The reaction mixture was cooled to room temperature and separated on a 20% denaturing polyacrylamide gel containing 7 M urea. Blanks for the assay consisted of reaction mixture and 20 *μ*g protein specimens that were heat-inactivated by boiling at 95°C for 5 min before initiating the activity assay. The radioactively labeled DNA was visualized using Image-Quant software (Molecular Dynamics, USA). The OGG1 8-oxodG-repair activity was determined and expressed as the amount of radioactivity in the incised product band relative to the radioactivity in the entire lane.

### 2.12. Immunoblotting and Immunoprecipitation

Mitochondrial OGG1 and SIRT3 protein content was determined on isolated mitochondria lysate, and A*β*-42 peptides lever was determined on whole hippocampus lysate. Proteins were separated by sodium dodecyl sulfate polyacrylamide gel electrophoresis using a 15% (OGG1, SIRT3) or 12% (A*β*-42) polyacrylamide gel and were subsequently transferred to a polyvinylidene difluoride membrane. The membranes were blocked with nonfat milk in PBST and incubated with rabbit polyclonal OGG1 antibody (1 : 1000, Abcam Biotechnology Inc.), rabbit polyclonal SIRT3 antibody (1 : 1000, Abcam Biotechnology Inc.), rabbit polyclonal A*β*-42 antibody (1 : 500, Santa Cruz Biotechnology Inc.), rabbit polyclonal COXIV antibody (1 : 2000, Invitrogen Inc.), and rabbit polyclonal GAPDH antibody (1 : 10000, Cell Signaling Technology Inc.). Detection was performed using an enhanced chemiluminescence detection system (Santa Cruz Biotechnology Inc.) with a secondary horseradish peroxidase- (HRP-) conjugated donkey anti-rabbit antibody (Cell Signaling Technology Inc.). Autoradiographic signals were assessed using a Bio-Rad scanning densitometer. COXIV was used as internal control for the experiments with isolated mitochondria, whereas GAPDH was used for the whole hippocampus blots.

For immunoprecipitation (IP) assays, mitochondria were isolated with the addition of 10 mM nicotinamide and 1 *μ*M trichostatin A. Equal amounts of mitochondrial protein were rotated for 2 h (4°C) with anti-OGG1 (Abcam Biotechnology Inc.), and then protein A/G PLUS-Agarose (Santa Cruz Biotechnology Inc.) was added and the samples were rotated overnight (4°C). The following morning, agarose beads were washed four times with RIPA buffer at 4°C before antigens were eluted from bead complexes with equal volume 1x SDS buffer. Samples were boiled for 10 min and were then separated by SDS-PAGE and immunoblotted using acetyl-lysine antibody (1 : 1000, Cell Signaling Technology Inc.), OGG1 antibody (1 : 1000, Abcam Biotechnology Inc.), and MnSOD antibody (1 : 1500, Abcam Biotechnology Inc.).

### 2.13. Statistical Analysis

The data were analyzed with Two-way ANOVA followed by Bonferroni* post hoc* tests. All values are expressed as the mean ± standard deviation of the mean. The Statistical Package for the Social Sciences (SPSS, Inc., version 13.0) was used for all analyses. The significance level was set at *P* < 0.05.

## 3. Results

### 3.1. Behavioral Functions

**Figure 1:**
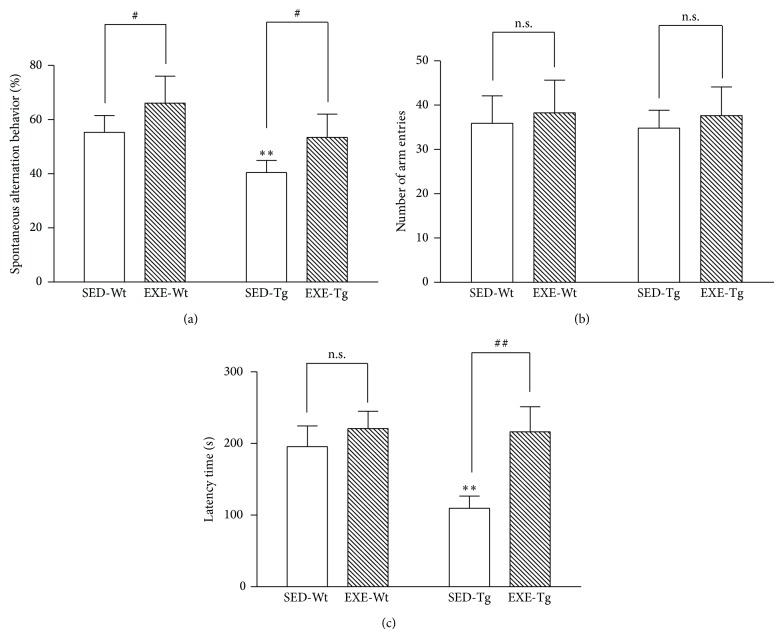
Effects of 20 weeks of treadmill exercise training on behavioral functions: (a) spontaneous alternation behavior; (b) total number of arm entries; (c) retention latency time. SED-Wt: sedentary wild-type mice. EXE-Wt: exercise-trained wild-type mice. SED-Tg: sedentary APP/PS1 transgenic mice. EXE-Tg: exercise-trained APP/PS1 transgenic mice. *n* = 32 for all independent experiments. Data are mean ± SD. ^**^ *P* < 0.01, compared to SED-WT group. ^#^ *P* < 0.05; ^##^ *P* < 0.01, compared to SED-Wt or SED-Tg group. n.s., nonsignificant.

In the SED groups, APP/PS1 Tg mice showed a significant reduction in spontaneous alternation behavior in the Y-maze test as compared to Wt mice (*P* < 0.01; Figure 1(a)). Following training, spontaneous alternation behavior was significantly increased in both Wt and Tg mice as compared to their respective controls in the SED groups (*P* < 0.05; Figure 1(a)). Moreover, the total number of arm entries was similar in all experimental groups (Figure 1(b)).

The retention latency time in the passive avoidance test was much lower in Tg mice than in Wt mice (*P* < 0.01; Figure 1(c)). In addition, compared to the sedentary animals, training significantly increased the retention latency time in Tg mice (*P* < 0.01; Figure 1(c)) but not in the WT mice.

### 3.2. A*β*-42 Levels

**Figure 2:**
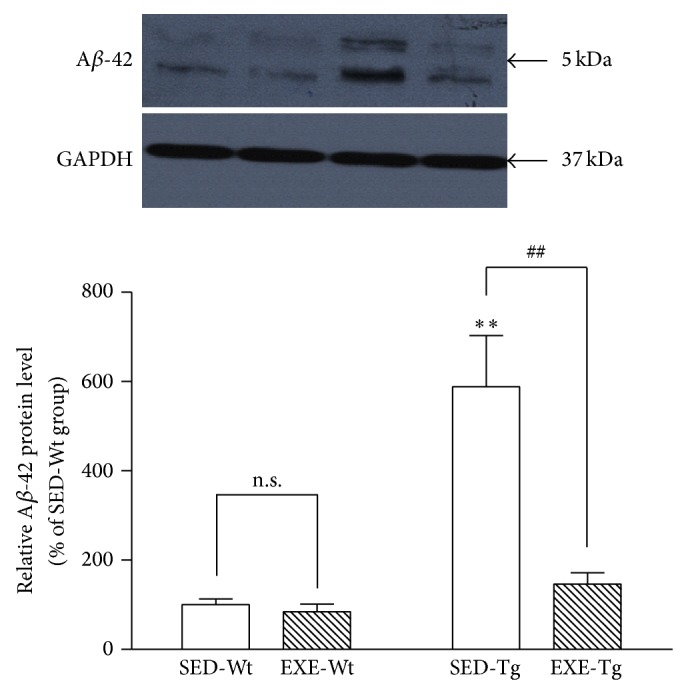
Effects of 20 weeks of treadmill exercise training on A*β*-42 protein level with Western blot. Group identities are the same as those in Figure 1. Hippocampus from four mice in one group was pooled for each sample, *n* = 8 for all independent experiments. Data are mean ± SD. ^**^ *P* < 0.01, compared to SED-WT group. ^##^ *P* < 0.01, compared to SED-Tg group. n.s., nonsignificant.

APP/PS1 Tg mice that were kept sedentary had a higher level of A*β*-42 than Wt mice (*P* < 0.01; Figure 2). However, training significantly decreased the level of A*β*-42 in Tg mice (*P* < 0.01; Figure 2). In the Wt mice, the level of A*β*-42 was not affected by training.

### 3.3. Mitochondrial Respiratory Function

**Figure 3:**
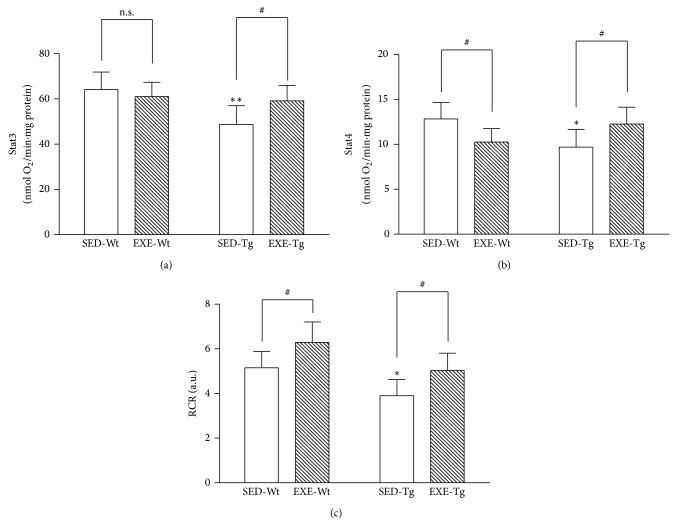
Effects of 20 weeks of treadmill exercise training on mitochondrial state 3 respiration rate (a), state 4 respiration rate (b), and respiratory control ratio (RCR) (c). Group identities are the same as those in Figure 1. Hippocampus from four mice in one group was pooled and mitochondria were isolated for each sample, *n* = 8 for all independent experiments. Data are mean ± SD. ^*^ *P* < 0.05; ^**^ *P* < 0.01, compared to SED-WT group. ^#^ *P* < 0.05, compared to SED-Wt or SED-Tg group. n.s., nonsignificant.

In the SED groups, APP/PS1 Tg mice showed significantly lower state 3 respiration rate, state 4 respiration rate, and RCR values compared to Wt mice (*P* < 0.05 or 0.01; Figure 3). Training elevated state 3 respiration rate, state 4 respiration rate, and RCR values in Tg mice (*P* < 0.05; Figure 3). In the Wt mice, exercise exerted no significant effect on state 3 respiration rate, while it significantly decreased state 4 respiration rate (*P* < 0.05; Figure 3(b)). As a result, mitochondrial RCR values were significantly increased in EXE-Wt group as compared to SED-Wt group (*P* < 0.05; Figure 3(c)).

**Figure 4:**
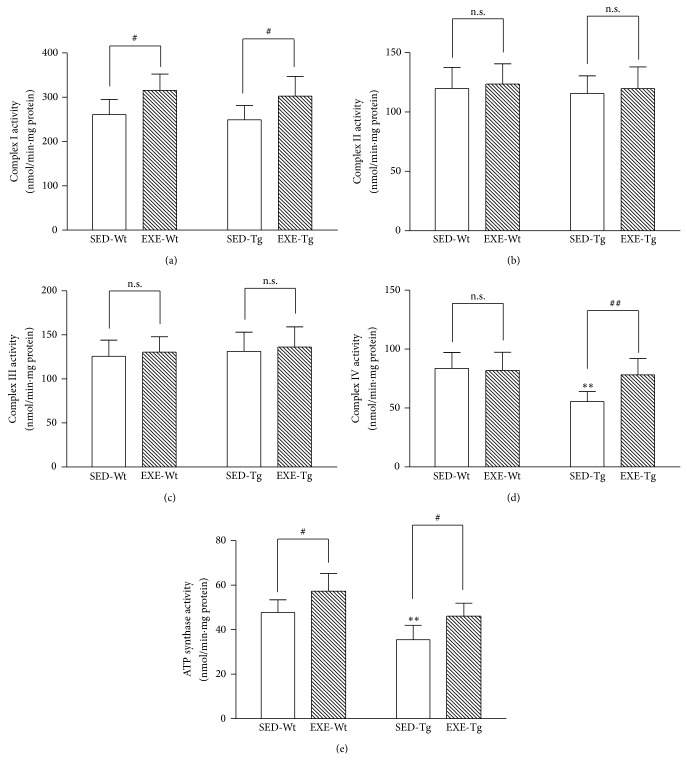
Effects of 20 weeks of treadmill exercise training on activities of mitochondrial complex I (a), complex II (b), complex III, (c), complex IV (d), and ATP synthase activity (e). Group identities are the same as those in Figure 1. Hippocampus from four mice in one group was pooled and mitochondria were isolated for each sample, *n* = 8 for all independent experiments. Data are mean ± SD. ^**^ *P* < 0.01 compared to SED-WT group. ^#^ *P* < 0.05; ^##^ *P* < 0.01, compared to SED-Wt or SED-Tg group. n.s., nonsignificant.

In the SED groups, the activities of complex IV and ATP synthase in the hippocampus were significantly decreased in APP/PS1 Tg mice as compared to Wt mice (*P* < 0.01; Figure 4), while the activities of complexes I, II, and III were similar between the two groups. Training elevated the activities of complexes I and IV and ATP synthase in Tg mice (*P* < 0.05 or 0.01; Figure 4). In the Wt mice, training significantly increased the activities of complex I and ATP synthase (*P* < 0.05; Figure 4).

### 3.4. Mitochondrial ROS Generation and 8-oxodG Content

**Figure 5:**
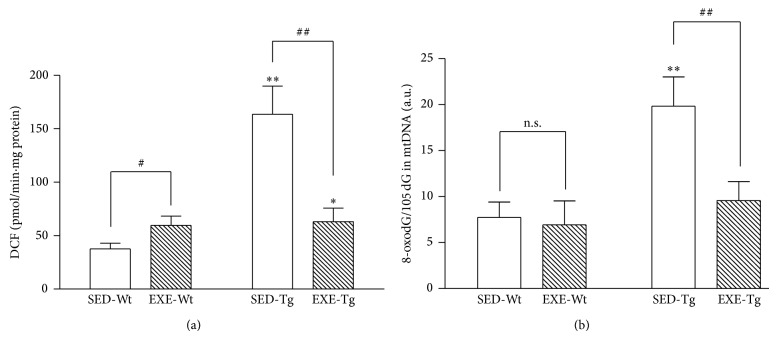
Effect of 20 weeks of treadmill exercise training on mitochondrial ROS generation (a) and 8-oxodG content of the mtDNA (b). Group identities are the same as those in Figure 1. Hippocampus from four mice in one group was pooled and mitochondria were isolated for each sample, *n* = 8 for all independent experiments. Data are mean ± SD. ^*^ *P* < 0.05; ^**^ *P* < 0.01, compared to SED-WT group. ^#^ *P* < 0.05; ^##^ *P* < 0.01, compared to SED-Wt or SED-Tg group. n.s., nonsignificant.

Sedentary APP/PS1 Tg mice showed markedly higher mitochondrial ROS generation rate (*P* < 0.01; Figure 5(a)) and 8-oxodG content (*P* < 0.01; Figure 5(b)) than Wt mice, whereas training significantly suppressed mitochondrial ROS generation rate and 8-oxodG content (*P* < 0.01; Figure 5). In the Wt mice, training elevated mitochondrial ROS production (*P* < 0.05; Figure 5(a)), while it exerted no effect on 8-oxodG content.

### 3.5. Mitochondrial Antioxidant Enzymes Activities

**Figure 6:**
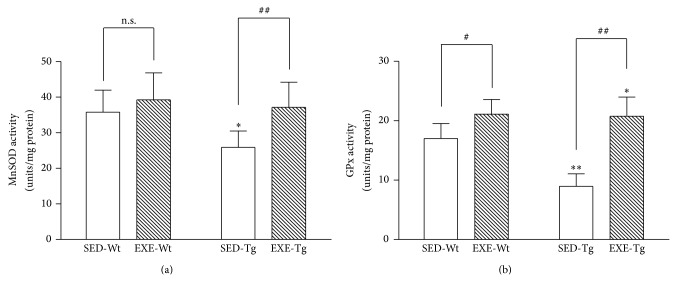
Effect of 20 weeks of treadmill exercise training on mitochondrial MnSOD (a) and GPx activity (b). Group identities are the same as those in Figure 1. Hippocampus from four mice in one group was pooled and mitochondria were isolated for each sample, *n* = 8 for all independent experiments. Data are mean ± SD. ^*^ *P* < 0.05; ^**^ *P* < 0.01, compared to SED-WT or SED-Tg group. ^#^ *P* < 0.05; ^##^ *P* < 0.01, compared to SED-Wt or SED-Tg group. n.s., nonsignificant.

In the SED groups, APP/PS1 Tg mice had significantly lower activities of mitochondrial GPx and MnSOD than Wt mice (*P* < 0.05 or 0.01; Figure 6). Training increased mitochondrial GPx and MnSOD activities in Tg mice (*P* < 0.01; Figure 6). In the Wt mice, only mitochondrial GPx activity was elevated with training (*P* < 0.05; Figure 6(b)), whereas no effect was observed in MnSOD activity.

### 3.6. Mitochondrial OGG1 Protein Level and Activity

**Figure 7:**
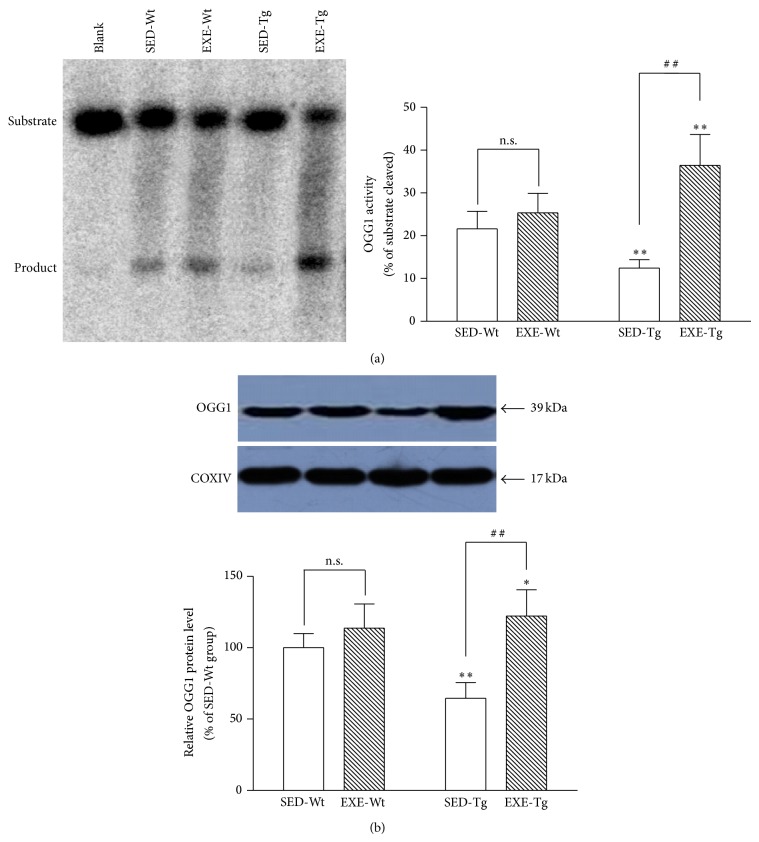
Effect of 20 weeks of treadmill exercise training on mitochondrial OGG1 activity (a) and protein content (b). Group identities are the same as those in Figure 1. Hippocampus from four mice in one group was pooled and mitochondria were isolated for each sample, *n* = 8 for all independent experiments. Data are mean ± SD. ^*^ *P* < 0.05, ^**^ *P* < 0.01, compared to SED-WT group. ^##^ *P* < 0.01, compared to SED-Wt or SED-Tg group. n.s., nonsignificant.

Mitochondrial OGG1 content and its 8-oxodG-repair activity were significantly lower in sedentary APP/PS1 Tg mice compared to Wt mice (*P* < 0.01; Figure 7). Training increased OGG1 content and its 8-oxodG-repair activity in Tg mice (*P* < 0.01; Figure 7) but not Wt mice.

### 3.7. Mitochondrial SIRT3 Expression and Acetylation Level of OGG1 and MnSOD

**Figure 8:**
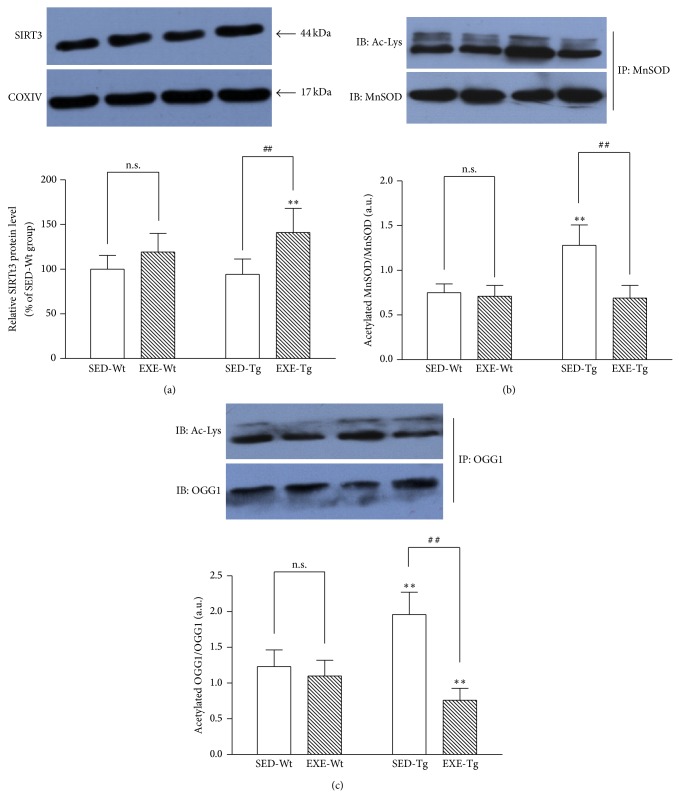
Effect of 20 weeks of treadmill exercise training on mitochondrial SIRT3 protein content (a), mitochondrial OGG1 acetylation level (b), and MnSOD acetylation level (c). Group identities are the same as those in Figure 1. Hippocampus from four mice in one group was pooled and mitochondria were isolated for each sample, *n* = 8 for all independent experiments. Data are mean ± SD. ^**^ *P* < 0.01, compared to SED-WT group. ^##^ *P* < 0.01, compared to SED-Wt or SED-Tg group. n.s., nonsignificant.

In the SED groups, there were no significant differences in mitochondrial SIRT3 content between APP/PS1 Tg mice and Wt mice. Training increased mitochondrial SIRT3 content in Tg mice (*P* < 0.01; Figure 8(a)) but not Wt mice. Immunoprecipitation assays showed that the acetylation level of mitochondrial OGG1 and MnSOD was significantly higher in sedentary APP/PS1 Tg mice compared to Wt mice (*P* < 0.01; Figure 8). Training decreased the acetylation of mitochondrial OGG1 and MnSOD in Tg mice (*P* < 0.01; Figure 8) but not Wt mice.

## 4. Discussion

### 4.1. Exercise Training Improves Mitochondrial Bioenergetics and Ameliorates the Pathological Phenotypes of AD 

In the present study, twenty weeks of treadmill exercise improved the cognition function of APP/PS1 Tg mice tested by passive avoidance and Y-maze test. The passive avoidance test assesses long-term learning and memory ability by allowing mice to form an association between escaping from an aversive stimulus (light) and a foot shock. Y-maze test has been used as relatively rapid procedures to assess short-term memory and space perception ability in rodents. This test is based on the natural tendency of rodents to alternate in their choices of arms visited when they are placed in a multiarm maze. Importantly, both of these tests are well known to be dependent on intact hippocampal function, evidenced by the deficits observed in hippocampectomised rodents [21].

The cybrid approaches revealed that mitochondrial deficiencies could be the origin of the increased oxidative stress and A*β* deposition found in AD brains. Studies in which “AD cybrid” lines were generated through the transfer of AD platelet mitochondria to endogenous mtDNA-depleted cells observed decreased ATP and increased oxidative stress and A*β* levels [5]. In the present study, we found that APP/PSI mice demonstrated a uniform impairment of mitochondrial function as indicated by decreased states 3 and 4 respiration rates and a reduction of RCR. These data indicate that mitochondrial dysfunction may play an important role in the etiology of this AD model. Furthermore, twenty weeks of treadmill exercise largely restored the observed mitochondrial deficit reflected by increased state 3 and 4 respiration and RCR and reduced A*β*-42 protein level in the hippocampus of APP/PS1 Tg mice. These effects may impact cognitive functions in exercised Tg mice, as indicated by the improved long-term memory in the passive avoidance test and spatial working memory in the Y-maze test.

Steiner et al. [22] have shown that 8 weeks of treadmill running elevated contents of sirtuin 1 (SIRT1) and peroxisome proliferator-activated receptor- (PPAR-) *γ* coactivator- (PGC-) 1*α*, which control the transcription of genes involved in mitochondrial oxidative phosphorylation in hippocampus. This finding is especially important because SIRT1 and PGC-1*α* expression are known to diminish in AD, which are correlated with mitochondrial dysfunction [23]. It has been shown that SIRT1 suppresses A*β* production by activating the *α*-secretase gene ADAM10 [24]. Recently, Wang et al. [25] demonstrated that modest fasting in mice could reduce *β*-secretase transcription in the brains through elevated PGC-1*α* expression and activity. It is known that exercise could activate the enzymes which degrade amyloid-*β*, such as neprilysin and proteasome [26]. Nevertheless, the present results suggest that training-induced inhibition of A*β*-42 generation might be partially mediated through improved mitochondrial function.

Brain-derived neurotrophic factor (BDNF) is closely implicated in exercise-induced enhancement of brain plasticity. Enhanced BDNF in the hippocampus is known to improve both short-term and long-term memories and contribute to neuronal survival and differentiation [27]. Recently, Wrann et al. [28] found that endurance exercise induces hippocampal BDNF through a PGC-1*α*/FNDC5 pathway. As such, exercise-induced BDNF may provide an important link between improved mitochondrial function and enhanced cognitive function. Interestingly, FNDC5 (fibronectin type III domain containing 5) in muscle and liver cells is cleaved, resulting in a protein fragment termed irisin that is released into the blood and enters the brain to induce BDNF expression in neurons. Thus, the effects of exercise on neuroplasticity are mediated, in part, by BDNF induced by local changes in the brain and by signals from peripheral tissues including muscle and liver.

### 4.2. Exercise Training Protection against mtDNA Oxidative Damage and Role of Mitochondrial OGG1

The mitochondrial genomes are vulnerable to free radical attack and the resultant oxidative modification of mtDNA is a known contributing factor neurodegeneration [7]. If oxidatively modified nucleosides such as 8-oxodG are not removed, they have the potential to mispair with adenine, resulting in a G : C to T : A transversion. Brégeon et al. [29] showed that 8-oxodG could cause RNA polymerase slippage resulting in a single mRNA base deletion leading to frameshift mutations of the transcript. Ballinger et al. [30] also reported that human umbilical vein endothelial cells treated with H_2_O_2_ displayed decreased levels of steady-state mRNA encoded by the mtDNA and reduced concentrations of all 13 polypeptides translated in the mitochondria.

Indeed, we showed that the activity of complex IV and ATP synthase in APP/PS1 Tg mice was markedly decreased compared to the Wt mice. However, the activities of these enzymes were significantly increased after endurance training. In AD, the most consistent defect in mitochondrial respiratory chain is found in COX activity, which is negatively correlated with A*β* concentration [31]. Mean COX activity was lower in cybrid line-containing mtDNA of AD subjects compared to the mtDNA of control subjects [5]. Manczak et al. [3] also reported that mtDNA-encoded ATPase 6 and ATPase 8 mRNA level were decreased among AD patients. These results suggest that mtDNA defects at least partly determine low complex IV and ATP synthase activity in AD. Based on these findings, it seems reasonable to suggest that the training-induced amelioration of ETC complexes (I, IV, and ATP synthase) in the APP/PS1 mice could be attributed to reduced 8-oxodG content in the mtDNA.

At steady state, the extent of mtDNA damage is determined by the oxidative modification of its base-pairs and the efficiency of the repair. The brain in particularly relies on DNA repair mechanisms due to a high level of oxygen consumption, a large number of mitochondria, and less antioxidant capacity than other tissues [8]. It has been shown that the activity and protein levels of BER enzymes are inversely correlated with the number of neurofibrillary tangles, which is associated with cognitive decline [32]. In addition, Iida et al. [9] used immunohistochemical methods to show that levels of the mitochondrial form of OGG1 were significantly decreased in the orbitofrontal cortex in the subjects with late-stage AD compared to that in normal subjects. These data were consistent with our finding that APP/PS1 mice had significantly lower OGG1 protein level and in vitro 8-oxodG-repair activity in the hippocampal mitochondria than the Wt mice. Thus, maintenance of an optimal level of OGG1 appears to be critical in protecting the integrity of mtDNA, shown by several previous studies using various experimental cell lines relevant to AD. For example, Panduri et al. [33] found that overexpression of mitochondria-targeted OGG1 could prevent oxidant-induced mitochondrial dysfunction in human lung adenocarcinoma cells by preserving mitochondrial aconitase, inactivation of which is known to promote superoxide radical from its (4Fe-4S)^2+^ center. Lee et al. [34] reported that overexpression of mitochondrial OGG1 effectively recovered mitochondrial respiratory function and decreased ROS generation in the SNU423 cells, which have low mitochondrial OGG1 activity. A novel finding in the present study was that exercise training increased both OGG1 protein content and its 8-oxodG repairing activity in the hippocampal mitochondrial of APP/PS1 Tg mice. This adaptation may play an important role in accounting for the improved mitochondrial energy metabolism, lower ROS generation, and mtDNA oxidative damage in these animals.

### 4.3. Potential Mechanism Involved in Exercise-Induced Upregulation of Mitochondrial OGG1

Previous research indicated that OGG1 expression and activity could be influenced by intracellular redox status controlled by ROS generation and antioxidant defense capacity. While oxidative stress can modify critical cysteine residues that must be kept in the reduced state for optimal OGG1 activity, a reduced mitochondrial glutathione (mtGSH) redox environment is deemed important as a posttranslational mechanism for keeping mtOGG1 active [35, 36]. In addition, an association between MnSOD and OGG1 protein-protein interactions might be important in the preservation of OGG1's DNA repairing activity [37]. Recently, Singh et al. [38] reported that antioxidant vitamin C and butylated hydroxyanisole supplement elevated OGG1 expression through induction of transcription factor nuclear factor erythroid 2-related factor 2 (Nrf2), which plays a crucial role in mediating expression of a series of antioxidant enzymes, such as SOD, GPx, and heme oxygenase-1. Since trained Tg mice demonstrated higher MnSOD and GPX activities and decreased ROS generation in present study, it is possible that mitochondrial OGG1 activity was ameliorated through modulation of mitochondrial redox status.

Sirtuin 3 (SIRT3), which is primarily localized in the mitochondrial matrix, targets various mitochondrial proteins for lysine deacetylation and regulates mitochondrial bioenergetics and biogenesis [39]. Sirt3 also promotes mitochondrial oxidative stress resistance via altering the acetylation level of MnSOD and enhancing its ability to scavenge ROS [40]. In our study, exercise training increased SIRT3 protein content and reduced MnSOD acetylation in the hippocampal mitochondrial of APP/PS1 Tg mice. These may play an important role in training-induced upregulation of mitochondrial antioxidant enzymes. Recently, Cheng et al. [41] demonstrated that SIRT3 was also physically associated with OGG1 and deacetylated this DNA glycosylase thereby preventing the degradation of the OGG1 and thus enhancing its 8-oxodG-repairing activity in the mitochondria. In the present study, we found mitochondrial OGG1 acetylation to be decreased in Tg mice after exercise training, in parallel with significantly increased level and activity of OGG1. This agrees with the hypothesis that SIRT3 deacetylase function is required for training-induced adaptation of mitochondrial OGG1.

## 5. Conclusion

**Figure 9:**
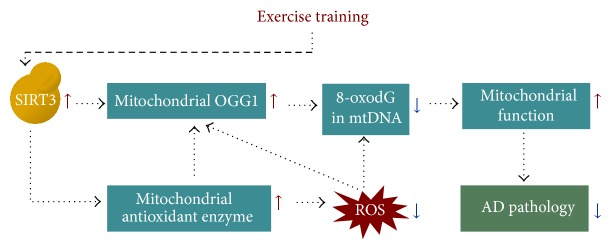
Possible mechanism of exercise training-induced neuroprotective effects in Alzheimer's disease (AD).

In summary, data presented in this study demonstrate that 20 weeks of treadmill exercise prevented decline of cognitive function, accumulation of A*β* level, and mitochondrial dysfunction, along with decreased mtDNA oxidative damage and increased mitochondrial respiratory capacity in the hippocampus of AD transgenic mice. Furthermore, increased OGG1 protein content and its 8-oxodG repairing activity in the hippocampal mitochondria were observed, which might be due to increased SIRT3 deacetylase function. Upregulation of mitochondrial OGG1 may also be influenced by decreased ROS generation and increased antioxidant enzymes activities. These findings suggest that exercise training could increase mtDNA repair capacity in the mouse hippocampus, which in turn would result in protection against mitochondrial dysfunction and phenotypic deterioration shown in the AD mice (Figure 9).
